# Pharmacokinetics, Safety and Efficacy of Intravenous Vedolizumab in Paediatric Patients with Ulcerative Colitis or Crohn’s Disease: Results from the Phase 2 HUBBLE Study

**DOI:** 10.1093/ecco-jcc/jjac036

**Published:** 2022-03-17

**Authors:** Jeffrey S Hyams, Dan Turner, Stanley A Cohen, Erzsébet Szakos, Kinga Kowalska-Duplaga, Frank Ruemmele, Nicholas M Croft, Bartosz Korczowski, Promise Lawrence, Siddharth Bhatia, Harisha Kadali, Chunlin Chen, Wan Sun, Maria Rosario, Senthil Kabilan, William Treem, Guillermo Rossiter, Richard A Lirio

**Affiliations:** Connecticut Children’s Medical Center, Hartford, CT, USA; Shaare Zedek Medical Center, The Hebrew University of Jerusalem, Jerusalem, Israel; Children’s Center for Digestive Health Care, Atlanta, GA, USA; Borsod-A-Z County Central University Teaching Hospital, Velkey Laszlo Paediatric Health Centre, University of Miskolc, Miskolc, Hungary; Department of Paediatrics, Gastroenterology and Nutrition, Jagiellonian University Medical College, Kraków, Poland; Université de Paris, APHP, Hôpital Necker Enfants Malades, Paediatric Gastroenterology, Paris, France; Centre for Immunobiology, Blizard Institute, Barts and the London School of Medicine, Queen Mary University of London and The Royal London Children’s Hospital, Barts Health NHS Trust, London, UK; Department of Paediatrics and Paediatric Gastroenterology, University of Rzeszów, Rzeszów, Poland; Takeda, Cambridge, MA, USA; Takeda, Cambridge, MA, USA; Takeda, Cambridge, MA, USA; Takeda, Cambridge, MA, USA; Takeda, Cambridge, MA, USA; Takeda, Cambridge, MA, USA; Takeda, Cambridge, MA, USA; Takeda, Cambridge, MA, USA; Takeda, Cambridge, MA, USA; Takeda, Cambridge, MA, USA

**Keywords:** Endoscopy, clinical trials, paediatrics

## Abstract

**Background and Aims:**

To date, there are no systematic pharmacokinetic [PK] data on vedolizumab in paediatric inflammatory bowel disease [IBD]. We report results from HUBBLE, a dose-ranging, phase 2 trial evaluating the PK, safety and efficacy of intravenous vedolizumab for paediatric IBD.

**Methods:**

Enrolled patients [aged 2–17 years] with moderate to severe ulcerative colitis [UC] or Crohn’s disease [CD] and body weight ≥10 kg were randomized by weight to receive low- or high-dose vedolizumab [≥30 kg, 150 or 300 mg; <30 kg, 100 or 200 mg] on Day 1 and Weeks 2, 6 and 14. Week 14 assessments included PK, clinical response and exposure–response relationship. Safety and immunogenicity were assessed.

**Results:**

Randomized patients weighing ≥30 kg [UC, *n* = 25; CD, *n* = 24] and <30 kg [UC, *n* = 19; CD, *n* = 21] had a baseline mean [standard deviation] age of 13.5 [2.5] and 7.6 [3.2] years, respectively. In almost all indication and weight groups, area under the concentration curve and average concentration increased ~2-fold from low to high dose; the trough concentration was higher in each high-dose arm compared with the low-dose arms. At Week 14, clinical response occurred in 40.0–69.2% of patients with UC and 33.3–63.6% with CD in both weight groups. Clinical responders with UC generally had higher trough concentration vs non-responders, while this trend was not observed in CD. Fourteen per cent [12/88] of patients had treatment-related adverse events and 6.8% [6/88] had anti-drug antibodies.

**Conclusions:**

Vedolizumab exposure increased in an approximate dose-proportional manner. No clear dose–response relationship was observed in this limited cohort. No new safety signals were identified.

## Graphical Abstract

**Graphical Abstract:**
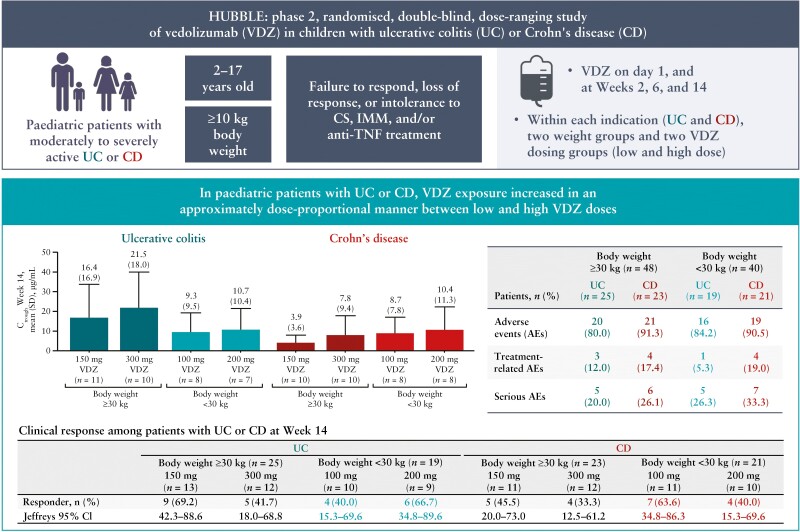

## 1. Introduction

Paediatric ulcerative colitis [UC] and Crohn’s disease [CD] share many features with the adult forms of the illnesses. However, children with UC and CD also have age-related complications related to growth, development, nutrition, bone mineral density accretion and psychosocial needs.^1^ Unlike biological therapy in adults, where weight-based dosing is linear, in young children with a body weight <30 kg, a higher dose per kilogram may be required to achieve similar trough concentration levels.^2^ This highlights the importance of dose-ranging studies in paediatric inflammatory bowel disease [IBD] to determine the optimal dose in all paediatric age groups.

The IBD treatment landscape has improved for children and adults since the development of biological therapy world-wide. In the USA, for example, several biologics are approved by the US Food and Drug Administration [FDA] for adults, two of which are also available for children. Intravenous [IV] infliximab [REMICADE, Janssen Biotech, Inc.] was the first to receive FDA approval for adults with UC and CD, and later for paediatric patients with IBD;^3^ subsequently, subcutaneous [SC] adalimumab [HUMIRA, AbbVie, Inc.] was FDA-approved for both adult and paediatric patients with UC and CD.^4^ IV and SC ustekinumab [STELARA, Janssen Biotech, Inc.] are FDA-approved for use only in adult, but not paediatric patients with UC and CD [currently in phase 3 trials in paediatric patients with CD and UC].^5^ Oral tofacitinib [XELJANZ, Pfizer, Inc.] and SC golimumab [SIMPONI, Janssen Biotech] are both FDA-approved for adult patients with UC, whereas phase 3 trials in paediatric patients with UC are ongoing.^6,7^ IV vedolizumab [ENTYVIO, Takeda Pharmaceuticals U.S.A., Inc.] is indicated for adults with moderately to severely active UC and CD,^8^ and paediatric phase 3 trials in UC^9^ and CD^10^ are now underway. Real-world studies evaluating off-label use of biologics, such as ustekinumab^11,12^ and vedolizumab,^13–15^ in the paediatric population suggest positive outcomes, but the safety and efficacy findings await confirmation from clinical trial data.

In previous adult studies of IV vedolizumab, positive exposure–response relationships for clinical response and remission were demonstrated, and these relationships appeared to be steeper in patients with UC than in those with CD.^16^ However, an effective and safe dose of IV vedolizumab in children has yet to be determined. Data from a previously developed population pharmacokinetic [PK] model based on vedolizumab phase 3 study results in adult patients with UC or CD were used to select the dose and weight groups evaluated in this paediatric study.^17^ Here, we now report results of HUBBLE, a paediatric phase 2 study of IV vedolizumab, a gut-selective monoclonal anti-α_4_β_7_-integrin antibody evaluating PK, safety and efficacy of IV vedolizumab in paediatric patients with UC and CD.

## 2. Methods

### 2.1. Study population

This study enrolled male and female paediatric patients [aged 2–17 years; body weight ≥10 kg] with moderate to severe UC or CD who failed to respond or were intolerant to corticosteroids, immunomodulators and/or tumour necrosis factor [TNF] antagonists. Moderately to severely active UC was defined as a complete Mayo score of 6–12, a total of Mayo subscores of stool frequency and rectal bleeding of ≥4, and an endoscopy subscore of ≥2.^18^ Moderately to severely active CD was defined as a simple endoscopic score for CD [SES-CD] of ≥7, the CD activity index [CDAI] components of average daily abdominal pain score of >1, and total number of liquid/very soft stools >10 for the 7 days before the first dose of study drug.^19,20^ Patients were excluded for previous exposure to approved or investigational anti-integrins, positive progressive multifocal leukoencephalopathy [PML] subjective symptom checklist, and current or anticipated surgical intervention for UC or CD. See the Supplementary Methods for complete inclusion and exclusion criteria.

### 2.2. Study design

HUBBLE [NCT03138655; EudraCT #2017-002231-41] was an international, phase 2, randomized, dose-ranging study. Patients with body weight ≥30 kg were enrolled at nine US and 14 non-US sites, and those weighing <30 kg were enrolled at seven US and 16 non-US sites. Patients were randomized 1:1 by body weight to receive a low or high IV vedolizumab dose [≥30 kg, 150 or 300 mg; <30 kg, 100 or 200 mg] on Day 1 and at Weeks 2, 6 and 14 [Supplementary Figure 1]. An interactive web response system was used for patient randomization and for dispensing medication ID number of the investigational drug. The high and low doses were selected on the basis of simulations performed using a population PK model derived from IV vedolizumab adult phase 3 data.^17^ Randomization was stratified by previous exposure/failure to TNFα antagonist therapy or naïvety to TNFα antagonist therapy, by indication [UC or CD] and by body weight group [≥30 or <30 kg]. Patients randomized to low-dose IV vedolizumab who did not show any clinical response were escalated to a high dose at Week 14. At Week 22, patients with clinical response could continue vedolizumab treatment in an extension study.

Oral 5-aminosalicylic acid [5-ASA], immunomodulators and corticosteroids [CS] were permitted during the study. Oral 5-ASA and immunomodulators had to be maintained at a constant dose throughout the first 14 weeks of the study. CS could be maintained at a maximum dose of ≤50 mg/day of prednisone [or equivalent steroid] or could be tapered down from the maximum dose under a specific protocol [see Supplementary Methods for more details on permitted medications and the CS tapering protocol].

### 2.3. Study endpoints and assessments

#### 2.3.1. PK and immunogenicity

The primary endpoints evaluated were PK parameters at Week 14 including area under the serum concentration curve [AUC], average concentration [*C*_avg_] and observed serum concentration at the end of the dosing interval [*C*_trough_]. Blood samples for PK assessment were collected post-dose on Day 1 and at Weeks 1 and 2, between Weeks 2 and 6, at Weeks 6, 10, 14 and 22, and at the final safety visit 18 weeks after the last dose of the study.

Vedolizumab serum concentrations were measured using a validated sandwich enzyme-linked immunosorbent assay, as previously described.^17^ Immunogenicity of vedolizumab was measured on the basis of the development of anti-drug antibodies [ADAs]. Blood samples were obtained within 30 min before dosing on Day 1 and at Weeks 2, 6, 14 and 22/early termination visit, and at any unscheduled visit for a patient who experienced a serious adverse event [AE] or disease exacerbation. Serum titres of ADAs were determined using a validated drug-tolerant electrochemiluminescence assay.^21^

#### 2.3.2. Efficacy

Disease activity was assessed at screening and during the study for efficacy using the Mayo and paediatric UC activity index [PUCAI] scores in UC, and the CDAI, paediatric CD activity index [PCDAI] and SES-CD scores in CD.^18–20^ For the clinical components of the scores, patients with UC or CD, or their parents/legal guardians made daily entries into a diary, and laboratory results [CD only] were collected during screening and the study period. Flexible sigmoidoscopy/colonoscopy assessed by a central reader was used to assess the endoscopic component of the Mayo score in patients with UC and the SES-CD score in patients with CD.

Secondary efficacy endpoints included the proportion of patients with UC who achieved clinical response at Week 14—defined as a reduction in complete Mayo score of ≥3 points and ≥30% reduction from baseline, with an accompanying decrease in rectal bleeding subscore of ≥1 point or absolute rectal bleeding subscore of ≤1 point—and in CD as a ≥70-point decrease from baseline in CDAI score.

Exploratory efficacy endpoints at Week 14 included: enhanced clinical response rate based on CDAI [≥100-point decrease from baseline in score]; clinical response rate based on a composite of SES-CD [≥50% reduction from baseline in score or score of 0–2] plus CDAI [decrease in average daily abdominal pain score of >0.25]; clinical response rate based on paediatric measures of PUCAI [≥20-point decrease from baseline in score] and PCDAI [≥15-point decrease from baseline in score and maximum total score of ≤30]; clinical remission rates based on each of the adult and paediatric measures [see definitions of clinical remission in Supplementary Methods]; and mean changes from baseline in faecal calprotectin.

#### 2.3.3. Vedolizumab exposure–response relationship

The IV vedolizumab exposure–response relationship was assessed at Week 14. This relationship was explored by evaluating *C*_trough_ by clinical response in each treatment group, for each indication and weight group separately. *C*_trough_ by clinical responsiveness was defined as a ≥3-point decrease and ≥30% reduction from baseline in complete Mayo score for UC, and a ≥70-point decrease from baseline in CDAI score for CD. According to paediatric measures, *C*_trough_ by clinical responsiveness was defined as a ≥20-point decrease from baseline in PUCAI score for UC and ≥15-point decrease from baseline in PCDAI score for CD.

#### 2.3.4. Safety

Safety assessments of all AEs and serious AEs that occurred throughout the study were conducted. AEs were categorized according to the *Medical Dictionary for Regulatory Activities* [MedDRA; versions 22.0 and 23.0U].

### 2.4. Statistical analyses

#### 2.4.1. Overview

The full analysis set [FAS] and safety analysis set included all patients who received at least one dose of study drug according to the treatment they were randomized to receive or treatment that they actually received, respectively. The PK set was defined as all patients who received at least one dose of study drug and had at least one measurable concentration of vedolizumab. All statistical analyses were conducted using SAS version 9.2 or higher.

#### 2.4.2. Sample size calculation

The planned sample size was 80 patients including 40 with a body weight of ≥30 kg and 40 with a body weight of 10 to <30 kg. Randomization caps were implemented to ensure that the sample size for each dose regimen was a minimum of nine patients with UC and nine patients with CD per weight group. A sample size of nine patients was expected to have ≥80% power to establish 95% confidence intervals [CIs] that were between 60% and 140% of the geometric mean estimates for total clearance after IV vedolizumab administration for each dose, indication and weight group, assuming the inter-patient variability for clearance in the paediatric population was similar to that in the adult population [% coefficient of variation ≤36.6%]. The sample size was based on industry guidance and was not adjusted for anticipated dropouts.^22,23^ The planned sample size was intended to allow for descriptive analysis of exposure and efficacy for each indication and dosing group.

#### 2.4.3. Primary and secondary efficacy analyses

All proportion-based efficacy endpoints are summarized by indication [UC or CD], weight group and treatment group. The point estimate, 95% Jeffrey’s interval and 95% CI based on Clopper–Pearson method are presented. Patients with any component of a scale missing were considered to be non-responders for that particular endpoint, scale and time point.

#### 2.4.4. PK and immunogenicity analyses

Descriptive statistics were used to summarize serum concentrations of vedolizumab over time and all PK parameters according to dose and weight groups; *C*_trough_ was also summarized according to response status. The PK parameters were derived by compartmental and/or non-compartmental approaches. This study was not designed or powered for hypothesis testing or comparing different groups.

The proportions of patients who were confirmed as ADA positive (persistent [positive ADA on at least two consecutive samples] or transient) or negative were summarized by weight group and treatment group for each indication separately in the FAS. Missing ADA data were not imputed.

### 2.5. Data availability

The datasets, including the redacted study protocol, redacted statistical analysis plan and individual participants’ data supporting the results reported in this article, will be made available within 3 months from initial request, to researchers who provide a methodologically sound proposal. The data will be provided after its de-identification, in compliance with applicable privacy laws, data protection, and requirements for consent and anonymization. Data are available upon request via application at https://search.vivli.org.

### 2.6. Study ethics and consent

The patient, or the patient’s legally acceptable representative, signed and dated an informed consent/assent form and patient authorization form [if applicable] prior to entering the study. The study was approved by the Institutional Review Board [or equivalent] at each participating centre.

## 3. Results

### 3.1. Study population

There were no differences in demographic characteristics between patients with UC and CD [Table 1]. There were some apparent differences between dose groups in baseline disease characteristics but comparisons are limited by the small sample size [Table 2].

**Table 1.** Baseline demographic characteristics in patients with UC or CD

	UC				CD			
	Body weight ≥30 kg [*n* = 25]		Body weight <30 kg [*n* = 19]		Body weight ≥30 kg [*n* = 24]		Body weight <30 kg [*n* = 21]	
	150 mg VDZ [*n* = 13]	300 mg VDZ [*n* = 12]	100 mg VDZ [*n* = 10]	200 mg VDZ [*n* = 9]	150 mg VDZ [*n* = 12]	300 mg VDZ [*n* = 12]	100 mg VDZ [*n* = 11]	200 mg VDZ [*n* = 10]
Age, years								
Mean [SD]	12.4 [2.7]	13.9 [2.6]	7.0 [3.6]	8.0 [3.2]	13.4 [2.5]	14.3 [2.0]	7.4 [3.5]	8.1 [2.7]
Median	13.0	14.0	6.5	9.0	13.5	15.0	7.0	8.5
Age categories, *n* [%]								
Children, 2–11 years^a^	4 [30.8]	2 [16.7]	9 [90.0]	8 [88.9]	3 [25.0]	2 [16.7]	9 [81.8]	9 [90.0]
Adolescents, 12–17 years	9 [69.2]	10 [83.3]	1 [10.0]	1 [11.1]	9 [75.0]	10 [83.3]	2 [18.2]	1 [10.0]
Sex, *n* [%]								
Male	8 [61.5]	6 [50.0]	6 [60.0]	5 [55.6]	4 [33.3]	9 [75.0]	7 [63.6]	5 [50.0]
Female	5 [38.5]	6 [50.0]	4 [40.0]	4 [44.4]	8 [66.7]	3 [25.0]	4 [36.4]	5 [50.0]
Height, cm								
Mean [SD]	153.1 [17.2]	160.0 [13.7]	119.6 [23.0]	122.4 [18.8]	157.9 [15.4]	158.0 [10.1]	120.6 [20.9]	124.7 [13.9]
Median [range]	156.0 [128.0–176.1]	159.7 [138.3–185.4]	118.0 [82.7–146.0]	125.8 [84.9–143.0]	162.7 [134.3–182.5]	155.9 [141.9–181.5]	128.0 [83.5–146.0]	130.0 [96.5–137.6]
Body weight, kg								
Mean [SD]	46.2 [12.6]	54.0 [14.3]	22.4 [6.6]	23.2 [6.4]	51.5 [13.4]	45.9 [10.7]	22.1 [6.3]	23.4 [5.1]
Median [range]	47.6 [30.3–75.9]	52.9 [32.0–78.9]	22.7 [12.8–29.6]	25.4 [10.2–29.8]	47.7 [31.4– 79.0]	41.4 [33.9–68.0]	24.1 [12.0–29.9]	24.5 [14.3–30.0]

CD, Crohn’s disease; SD, standard deviation; UC, ulcerative colitis; VDZ, vedolizumab.

The youngest patient in the ≥30 kg category was aged 8 years at enrolment.

**Table 2.** Baseline disease characteristics in patients with [A] UC or [B] CD

A				
	UC			
	Body weight ≥30 kg [*n* = 25]		Body weight <30 kg [*n* = 19]	
Characteristic	150 mg VDZ [*n* = 13]	300 mg VDZ [*n* = 12]	100 mg VDZ [*n* = 10]	200 mg VDZ [*n* = 9]
Disease duration				
Mean [SD], years^a^	2.0 [2.0]	2.7 [2.4]	2.2 [2.7]	2.6 [2.7]
Faecal calprotectin				
Patients, *n*	13	11	10	9
>500 μg/g, *n* [%]	12 [92.3]	9 [75.0]	8 [80.0]	7 [77.8]
Median [range], μg/g	2517.0 [410.0–10 654.0]	1466.0 [147.0–2996.0]	1193.5 [48.0–5546.0]	1824.0 [10.0–10 685.0]
C-reactive protein				
Patients, *n*	13	12	10	9
Mean [SD], mg/L	2.5 [4.4]	6.2 [11.2]	8.2 [15.3]	6.9 [7.1]
Median [range], mg/L	0.4 [0.2–15.8]	1.9 [0.2–39.7]	2.3 [0.2–50.4]	3.3 [0.2–19.3]
Anti-TNF antagonist history, *n* [%]				
Naïve	8 [61.5]	6 [50.0]	8 [80.0]	7 [77.8]
Exposed/failed	5 [38.5]	6 [50.0]	2 [20.0]	2 [22.2]
Complete Mayo score				
Patients, *n*	13	11	10	8
Mean [SD]	7.4 [1.9]	8.5 [2.1]	8.9 [1.8]	9.0 [1.4]
Median [range]	8.0 [4.0–10.0]	8.0 [6.0–12.0]	8.5 [6.0–11.0]	9.0 [7.0–11.0]
PUCAI score				
Patients, *n*	13	12	10	9
Mean [SD]	46.5 [16.9]	50.4 [19.8]	56.0 [8.10]	54.4 [13.1]
Median [range]	50.0 [5.0–70.0]	42.5 [25.0–80.0]	55.0 [45.0–70.0]	50.0 [40.0–80.0]
Mayo endoscopic subscore				
Severe, score of 3, *n* [%]	2 [15.4]	5 [41.7]	6 [60.0]	4 [44.4]
Concomitant medication use,^b^*n*				
Corticosteroids	6	5	3	4
Immunomodulators	6	3	5	2
B				
	CD			
	Body weight ≥30 kg [*n* = 24]		Body weight <30 kg [*n* = 21]	
Characteristic	150 mg VDZ [*n* = 12]	300 mg VDZ [*n* = 12]	100 mg VDZ [*n* = 11]	200 mg VDZ [*n* = 10]
Disease duration				
Mean [SD], years^a^	3.7 [2.2]	4.4 [2.5]	2.0 [1.1]	2.2 [2.4]
Faecal calprotectin				
Patients, *n*	10	12	11	10
>500 μg/g, *n* [%]	8 [66.7]	10 [83.3]	10 [90.9]	8 [80.0]
Median [range], μg/g	1257.5 [408.0–10 737.0]	1421.0 [22.0–2922.0]	1495.0 [483.0–4578.0]	1155.0 [162.0–18 978]
C-reactive protein				
Patients, *n*	11	12	11	10
Mean [SD], mg/L	21.8 [32.0]	30.5 [33.0]	17.1 [40.3]	27.2 [46.5]
Median [range], mg/L	3.3 [0.2–103.2]	18.0 [0.2–99.5]	1.8 [0.2–135.7]	7.9 [0.2–142.0]
Anti-TNF agonist history, *n* [%]				
Naïve	3 [25.0]	3 [25.0]	6 [54.5]	4 [40.0]
Exposed/failed	9 [75.0]	9 [75.0]	5 [45.5]	6 [60.0]
CDAI total score				
Patients, *n*	10	11	11	10
Mean [SD]	213.9 [113.0]	250.7 [49.5]	258.2 [57.9]	220.2 [120.1]
Median [range]	163.8 [117.0–492.0]	271.0 [172.0–307.0]	265.8 [129.0–354.0]	203.7 [89.0–426.0]
PCDAI score				
Patients, *n*	11	12	11	9
Mean [SD]	28.4 [10.1]	35.0 [9.1]	32.5 [10.1]	30.0 [10.8]
Median [range]	30.0 [15.0–45.0]	36.3 [23.0–50.0]	35.0 [5.0–45.0]	27.5 [18.0–48.0]
SES-CD score				
Patients, *n*	11	12	11	8
Mean [SD]	12.9 [5.8]	16.6 [6.1]	11.2 [8.5]	13.9 [5.7]
Severe, score of >15, *n* [%]	4 [33.3]	6 [50.0]	3 [27.3]	3 [30.0]
Concomitant medication use,^b^*n*				
Corticosteroids	6	5	4	4
Immunomodulators	4	10	7	5

CD, Crohn’s disease; CDAI, Crohn’s disease activity index; PCDAI, paediatric Crohn’s disease activity index; PUCAI, paediatric ulcerative colitis activity index; SD, standard deviation; SES-CD, simple endoscopic score for Crohn’s disease; TNF, tumour necrosis factor; UC, ulcerative colitis; VDZ, vedolizumab.

Disease duration was defined as the date from first diagnosis to date of informed consent.

Concomitant medications were those given prior or during the screening period and ongoing at informed consent and/or post-baseline/Day 1.

**Figure 1.**
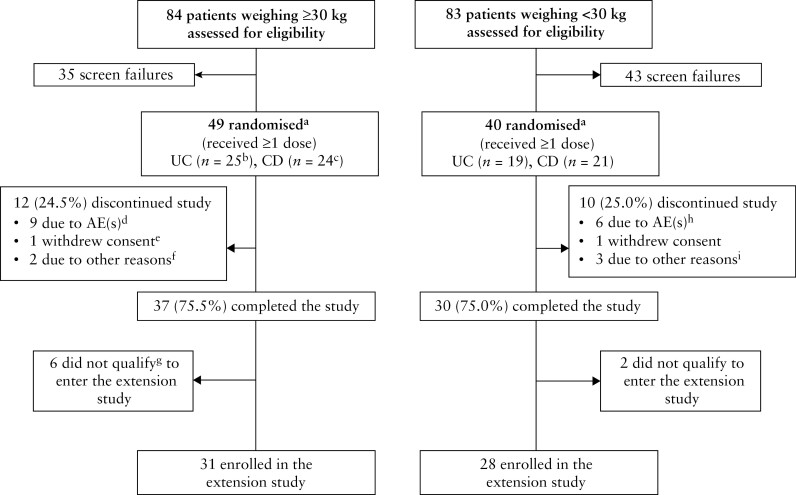
Patient disposition. AE, adverse event; CD, Crohn’s disease; TNF, tumour necrosis factor; UC, ulcerative colitis. ^a^Randomization stratified by previous exposure/failure to TNF antagonist therapy or naïvety to TNF antagonist therapy, by indication [UC or CD] and by weight group [≥30 kg, or 10 to <30 kg]. ^b^Includes one patient who received dose escalation to 300 mg vedolizumab at Week 14. ^c^Includes five patients who received dose escalation to 300 mg vedolizumab at Week 14. ^d^Worsening disease [*n* = 8] and lymphopenia considered associated with azathioprine use [*n* = 1]. ^e^This patient was randomized but not treated with study drug. ^f^Lack of efficacy [*n* = 1] and no improvement of disease [*n* = 1]. ^g^Based on clinical response criteria. ^h^Worsening disease [UC or CD; *n* = 5] and *Clostridium difficile* infection and procedural intestinal perforation in one patient with UC [*n* = 1]. ^i^Worsening disease and transition to other treatment option [*n* = 1], patient needed enteral nutrition [*n* = 1] and worsening CD [*n* = 1].

Patient disposition in the study is shown in Figure 1. Twelve [27.3%] of 44 patients with UC and ten [22.2%] of 45 patients with CD discontinued from the study. There was no difference in the discontinuation rate between weight groups. Compliance [number of completed infusions out of a total of four infusions] was 99% for patients weighing ≥30 kg and 98% for those <30 kg, with an overall mean (standard deviation [SD]) of 3.7 [0.7] and 3.7 [0.9] infusions completed [total amount of study drug infused] respectively.

### 3.2. PK and immunogenicity

**Figure 2.**
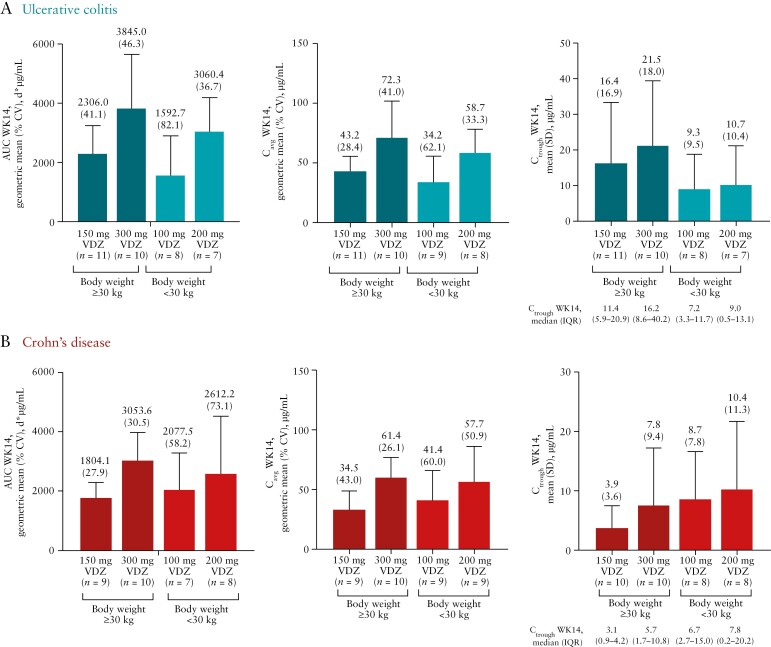
Pharmacokinetic parameters of VDZ AUC, *C*_avg_ and *C*_trough_ at Week 14 in patients with [A] ulcerative colitis and [B] Crohn’s disease [pharmacokinetic analysis set]. *N* = 88; one patient was randomized but not treated. The pharmacokinetic analysis set was defined as all patients who received at least one dose of VDZ and had at least one measurable concentration of VDZ. AUC, area under the serum concentration curve; *C*_avg_, average concentration; *C*_trough_, observed serum concentration at the end of the dosing interval; CV, coefficient of variation; IQR, interquartile range; SD, standard deviation; VDZ, vedolizumab; WK, Week.

In these paediatric patients with moderately to severely active UC and CD, vedolizumab serum exposure increased in an approximately dose-proportional manner for both weight groups. Consistently higher *C*_trough_ at Week 14 were observed in the high-dose groups, and Week 14 *C*_avg_ increased ~2-fold over the dose range from 150 to 300 mg in patients weighing ≥30 kg and from 100 to 200 mg in patients <30 kg [Figure 2]. Mean [SD] *C*_trough_ for all patients [combined CD and UC] was 10.4 [13.8] and 14.7 [15.7] µg/mL in patients weighing ≥30 kg and 9.0 [8.4] and 10.5 [10.5] µg/mL in those weighing <30 kg in the low- and high-dose groups, respectively.

In paediatric patients weighing ≥30 kg, vedolizumab Week 14 serum concentrations and derived PK parameters [particularly *C*_trough_ but less so AUC or *C*_avg_] were higher for patients with UC compared with patients with CD. This difference was not observed for patients weighing <30 kg.

Six [6.8%] of 88 patients were ADA-positive. Three patients [all with UC] were persistently ADA-positive [with two or more consecutive confirmed positive samples]. Among patients weighing ≥30 kg, 12.0% [3/25] with UC [all in the 150 mg group] were ADA-positive at any time during the study and 8.7% [2/23] with CD were ADA-positive [both patients were in the 300 mg group]. Among patients weighing <30 kg, 5.3% [1/19] with UC [100 mg group] were ADA-positive but no patients with CD were ADA-positive. Overall, there was no apparent trend suggesting that positive ADA status had a negative impact on PK parameters or achievement of clinical response at Week 14. However, interpretation of this is limited due to the small number of patients who were positive for ADA.

There was no trend showing that co-administration of immunomodulators was associated with a decrease in ADA rate in patients weighing ≥30 kg for both UC and CD. The impact of immunomodulators on immunogenicity in patients weighing <30 kg could not be evaluated because there was only one positive ADA case in this group.

### 3.3. Efficacy and exposure–response

According to adult measures, clinical response rates across weight and dose groups at Week 14 ranged from 40.0 to 69.2% in patients with UC [based on a reduction in complete Mayo score of ≥3 points and ≥30% from baseline] and from 33.3 to 63.6% in patients with CD [based on a ≥70-point decrease from baseline in CDAI score] [Table 3]. The enhanced clinical response rates [≥100-point decrease from baseline in CDAI score] at Week 14 ranged from 25.0% to 63.6% in patients with CD [Supplementary Table 1]. Clinical response [based on a composite of improvements in CDAI abdominal pain/stool subscores and SES-CD score] occurred at lower rates than the other clinical endpoints in patients with CD [Supplementary Table 2].

**Table 3.** Clinical response rates among patients with UC or CD [based on complete Mayo^a^ or CDAI^b^ scores] at Week 14 [full analysis set]

	UC				CD			
	Body weight ≥30 kg [*n* = 25]		Body weight <30 kg [*n* = 19]		Body weight ≥30 kg [*n* = 23]		Body weight <30 kg [*n* = 21]	
	150 mg VDZ [*n* = 13]	300 mg VDZ [*n* = 12]	100 mg VDZ [*n* = 10]	200 mg VDZ [*n* = 9]	150 mg VDZ [*n* = 11]	300 mg VDZ [*n* = 12]	100 mg VDZ [*n* = 11]	200 mg VDZ [*n* = 10]
Responder, *n* [%]	9 [69.2]	5 [41.7]	4 [40.0]	6 [66.7]	5 [45.5]	4 [33.3]	7 [63.6]	4 [40.0]
Jeffreys 95% CI^c^	42.3–88.6	18.0–68.8	15.3–69.6	34.8–89.6	20.0–73.0	12.5–61.2	34.8–86.3	15.3–69.6
Exact 95% CI^d^	38.6–90.9	15.2–72.3	12.2–73.8	29.9–92.5	16.7–76.6	9.9–65.1	30.8–89.1	12.2–73.8
Non-responder, *n* [%]	4 [30.8]	7 [58.3]	6 [60.0]	3 [33.3]	6 [54.5]	8 [66.7]	4 [36.4]	6 [60.0]
Jeffreys 95% CI^c^	11.4–57.7	31.2–82.0	30.4–84.7	10.4–65.2	27.0–80.0	38.8–87.5	13.7–65.2	30.4–84.7
Exact 95% CI^d^	9.1–61.4	27.7–84.8	26.2–87.8	7.5–70.1	23.4–83.3	34.9–90.1	10.9–69.2	26.2–87.8

The full analysis set included all patients who received at least one dose of study drug according to the treatment they were randomized to receive.

CD, Crohn’s disease; CDAI, Crohn’s disease activity index; CI, confidence interval; UC, ulcerative colitis; VDZ, vedolizumab.

Clinical response, based on the complete Mayo score, was defined as a reduction in complete Mayo score of ≥3 points and ≥30% reduction from baseline with an accompanying decrease in rectal bleeding subscore of ≥1 point or absolute rectal bleeding subscore of ≤1 point; calculation was based on the GEMINI approach. Any patient with missing data for determination of clinical response status was counted as a non-responder. Patients with missing data at Week 14: body weight ≥30 kg [*n* = 2 in 150 mg VDZ, *n* = 2 in 300 mg VDZ]; body weight <30 kg [*n* = 3 in 100 mg VDZ, *n* = 1 in 200 mg VDZ].

Clinical response, based on the CDAI score, was defined as a ≥70-point reduction from baseline in CDAI score. Any patient with missing data for determination of response status was counted as a non responder. Patients with missing data at Week 14: body weight ≥30 kg [*n* = 1 in 150 mg VDZ, *n* = 2 in 300 mg VDZ]; body weight <30 kg [*n* = 3 in 100 mg VDZ, *n* = 4 in 200 mg VDZ].

Calculated using Jeffreys method. Jeffreys interval is a Bayesian credible interval obtained using the non-informative Jeffreys prior.

The exact 95% CI was constructed on the basis of the Clopper–Pearson method.

According to paediatric measures, clinical response rates across weight and dose groups at Week 14 ranged from 50.0 to 80.0% in patients with UC [based on a ≥20-point decrease from baseline in PUCAI score] and from 45.5 to 54.5% in patients with CD [based on a ≥15-point decrease in PCDAI score] [Supplementary Table 3].

**Figure 3.**
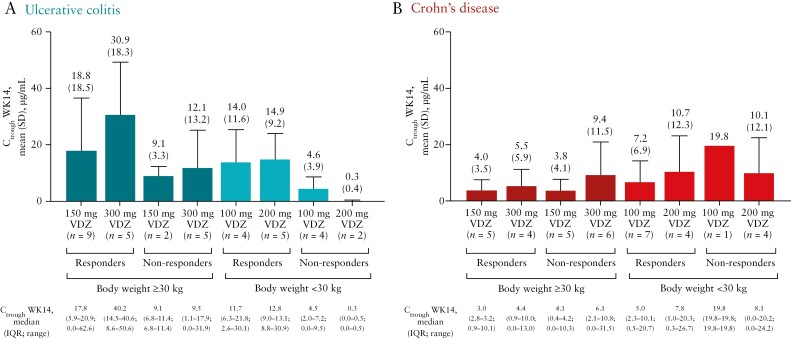
VDZ exposure at Week 14 according to clinical responsiveness in patients with [A] ulcerative colitis and [B] Crohn’s disease [pharmacokinetic analysis set]. The exposure–response relationship was based on reduction in complete Mayo score of ≥3 points and ≥30% from baseline for patients with ulcerative colitis and a ≥70-point decrease in Crohn’s disease activity index score from baseline for patients with Crohn’s disease. The pharmacokinetic analysis set was defined as all patients who received at least one dose of VDZ and had at least one measurable concentration of VDZ. *C*_trough_, observed serum concentration at the end of the dosing interval; IQR, interquartile range; SD, standard deviation; VDZ, vedolizumab; WK, Week.

In the evaluation of vedolizumab exposure–response, in patients with UC, generally a higher *C*_trough_ was observed in patients who achieved clinical response at Week 14 compared with patients who did not achieve clinical response in each dose arm according to complete Mayo and PUCAI scores. This trend was not observed in patients with CD, based on CDAI and PCDAI scores [adult measures are shown in Figure 3 and paediatric measures in Supplementary Table 4].

Clinical remission rates at Week 14 according to adult and paediatric measures are reported in Supplementary Tables 5 and 6, respectively. In patients with UC, clinical remission rates across weight and dose groups at Week 14 ranged from 20.0 to 38.5% on Mayo and from 30.0 to 61.5% on PUCAI. In patients with CD, clinical remission rates across weight and dose groups at Week 14 ranged from 50.0 to 63.6% on CDAI and from 16.7 to 54.5% on PCDAI. Faecal calprotectin levels at baseline were highly variable, with change during treatment seen across most dosing and weight groups in UC and CD [Supplementary Table 7].

### 3.4. Safety

The overall duration of exposure to vedolizumab was a median of 99.0 days in both weight groups with a range of 98.0–100.5 days. The incidence of AEs was similar between both disease indications in both weight groups: among patients with UC and CD, at least one AE was experienced by 80.0% and 91.3% of patients weighing ≥30 kg, respectively, and 84.2% and 90.5% of patients weighing <30 kg, respectively; most AEs were considered mild and not related to study drug [Table 4]. Of the 22 patients who discontinued, 15 discontinued due to AEs; of these, 13 were due to AEs of worsening UC or CD. Headache was the most commonly reported AE in the ≥30 kg group and abdominal pain was the most frequently reported AE in the <30 kg group. In patients with UC, headaches affected <10% of patients in the ≥30 kg group and 10.5% of patients in the <30 kg group. In patients with CD, headaches affected 21.7% of patients in the ≥30 kg group and <10% of patients in the <30 kg group. Abdominal pain affected <10% of patients with UC and CD in the ≥30 kg group and 21.1% of patients with UC and 28.6% of patients with CD in the <30 kg group [all AEs that occurred in ≥10% of patients are reported in Supplementary Table 8]. Most AEs of abdominal pain were mild to moderate in intensity and not related to study drug; they were possibly related to underlying disease pathology. Infections and infestations occurred in 48.0% of patients with UC and 47.8% of patients with CD in the ≥30 kg group (most were reported as viral infections [10.4% of total UC and CD patients] or ear infections, nasopharyngitis, pharyngitis or upper respiratory tract infection [6.3% each of total UC and CD patients]), and 36.8% and 28.6% in patients with UC and CD, respectively, in the <30 kg group [only upper respiratory tract infection, respiratory tract infection and viral infections were reported in more than one patient]. There were no cases of PML during the study.

**Table 4.** Overview of AEs^a^^,^^b^ among patients with UC or CD [safety analysis set]

Patients, *n* [%] [AEs, *n*]	Body weight ≥30 kg [*n* = 48]		Body weight <30 kg [*n* = 40]	
	UC [*n* = 25]	CD [*n* = 23]	UC [*n* = 19]	CD [*n* = 21]
AEs by relation to study drug	20 [80.0] [64]	21 [91.3] [106]	16 [84.2] [61]	19 [90.5] [90]
Related	3 [12.0] [4]	4 [17.4] [15]	1 [5.3] [1]	4 [19.0] [6]
Not related	17 [68.0] [60]	17 [73.9] [91]	16 [84.2] [60]	19 [90.5] [84]
AEs by severity				
Mild	9 [36.0] [41]	9 [39.1] [70]	10 [52.6] [24]	17 [81.0] [54]
Moderate	7 [28.0] [18]	7 [30.4] [21]	10 [52.6] [29]	9 [42.9] [28]
Severe	4 [16.0] [5]	5 [21.7] [15]	4 [21.1] [8]	5 [23.8] [8]
Leading to study drug discontinuation	1 [4.0] [1]	3 [13.0] [3]	3 [15.8] [4]	2 [9.5] [2]
Serious AEs	5 [20.0] [7]	6 [26.1] [12]	5 [26.3] [12]	7 [33.3] [13]
Related	0	1 [4.3] [1]^c^	0	0
Not related	5 [20.0] [7]	5 [21.7] [11]	5 [26.3] [12]	7 [33.3] 13]
Leading to study drug discontinuation	0	1 [4.3] [1]	3 [15.8] [3]	1 [4.8] [1]
Deaths	0	0	0	0

Relatedness, severity and seriousness of AEs are not mutually exclusive terms and patients could be counted in multiple categories. The safety analysis set was defined as all patients who received at least one dose of study drug according to the treatment they actually received.

AE, adverse event; CD, Crohn’s disease; UC, ulcerative colitis; VDZ, vedolizumab.

An AE was defined as an event whose date of onset occurred on or after the first dose of study drug through Week 22 for patients entering the extension study, or through the final safety visit 18 weeks after their last dose of study drug for those who did not enter the extension study.

Patients with one or more AEs within a level of *Medical Dictionary for Regulatory Activities* [version 23.0U] term were counted only once in that level.

A single serious AE of viral infection in a patient with CD in the dose-escalation group was considered related to vedolizumab treatment but no change was made to study medication.

Serious AEs occurred in 20% of patients with UC and 26.1% of patients with CD in the ≥30 kg group and 26.3% and 33.3%, respectively, in the <30 kg group [Table 4]. A single serious AE of viral infection in a patient with CD in the dose-escalation group was considered related to vedolizumab treatment, but no change was made to study medication. No deaths occurred during the study.

The criteria applied for the identification of hypersensitivity included standardized MedDRA queries of anaphylactic/anaphylactoid shock conditions [broad], angio-oedema [broad] and hypersensitivity [broad]. Among patients weighing ≥30 kg, ten [20.8%] reported hypersensitivity AEs. Although most were mild or moderate, there was one severe hypersensitivity AE [erythema nodosum] which was described as non-serious, unrelated to treatment, and not requiring any change to the dose of study medication, and two hypersensitivity AEs that were assessed as being related to treatment [asthma and hypersensitivity]. Among patients weighing <30 kg, hypersensitivity AEs were reported for four [10%], all of which were mild or moderate in intensity; none were assessed as being related to treatment. None of the hypersensitivity AEs were reported as serious AEs, and no cases of anaphylaxis or infusion-related reactions were reported in either cohort. One patient with hypersensitivity reactions was persistently positive for ADA and was positive for neutralizing ADA. There was no relationship between persistent ADA and hypersensitivity reaction.

## 4. Discussion

In this study, vedolizumab PK parameters were evaluated in a paediatric UC and CD population. The exposure in paediatric patients reported in this study [*C*_trough_ levels of 9.0–14.7 µg/mL] can be considered efficacious and within the range of therapeutic levels in adult patients after standard IV induction doses, based on PK data and modelling from previous adult phase 3 studies.^17,24,25^ In paediatric patients with moderately to severely active UC and CD, vedolizumab serum exposure increased in an approximately dose-proportional manner for both weight groups, and achieved concentrations similar to those of adults from the previous GEMINI trials. Consistently higher *C*_trough_ concentrations at Week 14 were observed in the high-dose groups.

In addition, vedolizumab serum concentrations and derived PK parameters at Week 14 were higher for paediatric patients weighing ≥30 kg with UC compared with patients with CD, whereas this difference was not observed for patients weighing <30 kg. However, there was variability in exposure observed across the study population, which was more apparent in *C*_trough_ than *C*_avg_. Indeed, the difference between these UC and CD groups was much more apparent for *C*_trough_ levels, whereas *C*_avg_ levels were generally more comparable, suggesting broadly similar PK characteristics between the patients with UC and CD.

The vedolizumab exposure–response relationship results showed that in patients with UC, *C*_trough_ at Week 14 was generally higher in responders than in non-responders in both treatment arms for both weight groups. However, due to the small sample size, these data do not clearly show that higher dosing in patients with UC was associated with superior clinical outcomes. In patients with CD, there was no clear trend for a higher *C*_trough_ in responders for both weight groups. No firm conclusion can be drawn regarding the exposure–response relationship in patients with UC or CD due to the small sample size. In patients with UC weighing ≥30 kg, response rates were generally greater in the low-dose vs high-dose group, with somewhat overlapping 95% CIs, whereas the high-dose group was favoured over the low-dose group in those weighing <30 kg. This may be due to slight differences in baseline disease characteristics such as higher baseline Mayo score, Mayo endoscopic subscore and C-reactive protein level in the high-dose group of patients with UC weighing ≥30 kg, as well as the higher proportion of patients with severe baseline PUCAI scores in the high-dose group. The baseline disease characteristics in patients with UC weighing <30 kg were generally similar between dose groups. There were no clear differences in efficacy among dose groups in the patients with CD.

The current paediatric UC population in this study and adult population in the GEMINI 1 study had similar disease severity and similar prior exposure to TNF antagonist drugs at baseline.^24^ However, with regard to CD, baseline CDAI scores were higher in adults [GEMINI 2]^25^ but baseline median faecal calprotectin and C-reactive protein levels were higher in the children in this study, and a greater proportion of children had been previously exposed to TNFα antagonist therapy. It is important to note that CDAI is a disease activity score for adults with CD, which makes translatability to the paediatric population difficult; however, this scoring index has also been previously used for regulatory purposes. It is interesting to compare the current results in children vs those previously reported for adults in the GEMINI trials, although it should be noted that vedolizumab exposure and clinical response were assessed at different time points in HUBBLE [Week 14] vs GEMINI 1 and 2 [Week 6]. Overall, the current PK parameters and the dose-proportional increase in serum concentration levels in paediatric patients were consistent with previous data shown in adults across the same dose range. Vedolizumab treatment with 150 and 300 mg IV doses [≥30 kg group] and 100 and 200 mg IV doses [<30 kg group] in paediatric patients with UC or CD resulted in similar clinical responses at Week 14 to those observed in adults in the previous phase 3 studies of IV vedolizumab [300 mg] during induction therapy [Week 6].^24,25^ In the phase 3 GEMINI 1 study, clinical response was achieved by 47.1% of adults with UC who were evaluated at Week 6 of induction therapy [300 mg IV vedolizumab]. In contrast, at Week 14 of the current study, clinical response was achieved in 56.0% of children with UC weighing ≥30 kg [150 and 300 mg IV vedolizumab] and in 52.6% of children with UC weighing <30 kg [100 and 200 mg IV vedolizumab].^24^ In the phase 3 GEMINI 2 study, a reduction of CDAI score by ≥100 points was achieved by 31.4% of adults with CD who were evaluated at Week 6 of induction therapy [300 mg IV vedolizumab].^25^ In contrast, at Week 14 of the current study, a reduction of CDAI score by ≥100 points was achieved in 30.4% of children with CD weighing ≥30 kg [150 and 300 mg IV vedolizumab] and in 47.6% of children with CD weighing <30 kg [100 and 200 mg IV vedolizumab]. Although fixed dosing in this phase 2 paediatric study appeared to be adequate compared with the adult trials, further study is needed on maintenance dosing strategies in the paediatric population, especially in the lowest weight group of <30 kg.

Vedolizumab was generally safe and well tolerated when administered at either 150 and 300 mg in paediatric patients weighing ≥30 kg or at 100 and 200 mg in patients weighing <30 kg, with a comparable safety profile in both weight groups. The overall safety profile observed in this study is generally consistent with the known safety profile of vedolizumab in adults and with the common symptoms of IBD.^24–26^

This study has some limitations. It was not designed or powered for comparing between-group differences in PK parameters, but for descriptive analysis of exposure and efficacy within each indication and dosing group. Any potential inaccuracies in pre-dose sampling time and in a study of this sample size would lead to variability in the PK parameters, in particular *C*_trough_ levels, although *C*_avg_ would be less affected. The small number of paediatric patients, especially in the youngest age group, precluded concrete interpretation of comparisons between disease indication, weight group and/or dose group for baseline characteristics, safety and clinical response. Additionally, Mayo and CDAI scores were not designed to assess clinical response in children; however, at the time of this phase 2 trial, these measures were recommended by regulatory agencies to enable comparison of paediatric results to previous adult data. Future paediatric IBD studies will probably use paediatric measures [PUCAI and PCDAI] or the SES-CD.

In conclusion, vedolizumab serum exposure increased in an approximately dose-proportional manner in this paediatric population, and vedolizumab exposure, clinical response and safety were considered similar in children relative to adult patients in historical studies. The results from the HUBBLE study, in conjunction with adult data from the GEMINI studies, have been used to determine ideal dosing schedules for further studies in paediatric patients.

## Supplementary Material

jjac036_suppl_Supplementary_Data

jjac036_suppl_Supplementary_Figure_S1

## Conference Presentation

Parts of these data were presented at the 2021 Digestive Disease Week, May 21–23, 2021, virtual congress, Poster Su497, and the 2021 World Congress of Pediatric Gastroenterology, Hepatology and Nutrition, June 2–5, 2021, virtual congress, Poster G-ePwP-064.
