# Supplementary material for: Pharmacokinetics, Safety and Efficacy of Intravenous Vedolizumab in Paediatric Patients with Ulcerative Colitis or Crohn’s Disease: Results from the Phase 2 HUBBLE Study
Source: J Crohns Colitis. 2022 Mar 17;16(8):1243–54. doi: 10.1093/ecco-jcc/jjac036 (PMC9426668; doi:10.1093/ecco-jcc/jjac036)
Supplement: jjac036_suppl_Supplementary_Data [file jjac036_suppl_supplementary_data.docx]

**Supplementary Data**

This appendix has been provided by the authors to give readers additional information about their work.

Supplement to: Pharmacokinetics, Safety and Efficacy of Intravenous Vedolizumab in Paediatric Patients with Ulcerative Colitis or Crohn’s Disease: Results from the Phase 2 HUBBLE Study

Jeffrey S. Hyams,^a^ Dan Turner,^b^ Stanley A. Cohen,^c^ Erzsébet Szakos,^d^ Kinga Kowalska-Duplaga,^e^ Frank Ruemmele,^f^ Nicholas M. Croft,^g^ Bartosz Korczowski,^h^ Promise Lawrence,^i^ Siddharth Bhatia,^i^ Harisha Kadali,^i^ Chunlin Chen,^i^* Wan Sun,^i^* Maria Rosario,^i^* Senthil Kabilan,^i^ William Treem,^i^ Guillermo Rossiter,^i^ Richard A. Lirio^i^*

^a^Connecticut Children's Medical Center, Hartford, CT, USA
^b^Shaare Zedek Medical Center, The Hebrew University of Jerusalem, Jerusalem, Israel ^c^Children's Center for Digestive Health Care, Atlanta, GA, USA
^d^Borsod-A-Z County Central University Teaching Hospital, Velkey Laszlo Paediatric Health Centre, University of Miskolc, Miskolc, Hungary
^e^Department of Paediatrics, Gastroenterology and Nutrition, Jagiellonian University Medical College, Kraków, Poland
^f^Université de Paris, APHP, Hôpital Necker Enfants Malades, Paediatric Gastroenterology, Paris, France

^g^Centre for Immunobiology, Blizard Institute, Barts and the London School of Medicine, Queen Mary University of London and The Royal London Children's Hospital, Barts Health NHS Trust, London, UK

^h^Department of Paediatrics and Paediatric Gastroenterology, University of Rzeszów, Rzeszów, Poland
^i^Takeda, Cambridge, MA, USA

*Employees of Takeda at the time of this research.

**Supplementary Methods**

**Complete study inclusion and exclusion criteria**

***Inclusion criteria***

Patient eligibility was determined according to the following criteria prior to entry into the study:

1. In the opinion of the investigator, the patient, parent or legal guardian was capable of understanding and complying with protocol requirements.
2. The patient and/or the parent or legal guardian gave voluntary informed consent/assent and signed and dated a written informed consent/assent form and any required privacy authorisation prior to the initiation of any study procedures.
3. The patient:
   1. was male or female; and
   2. weighed ≥10 kg at the time of randomisation; and
   3. was 2 to 17 years, inclusive, at the time of randomisation, with moderately to severely active ulcerative colitis [UC] or Crohn’s disease [CD] diagnosed at least 3 months prior to screening by clinical and endoscopic evidence and corroborated by a histopathology report.
4. The patient had a medical history of moderately to severely active UC during screening, defined as a complete Mayo score of 6–12, a total of Mayo subscores of stool frequency and rectal bleeding of ≥4, and an endoscopy subscore of ≥2; or had moderately to severely active CD defined as simple endoscopic score for CD [SES-CD] of ≥7, and the CD activity index [CDAI] components of average daily abdominal pain score of >1, and total number of liquid/very soft stools >10 for the 7 days prior to the first dose of study drug.
5. The patient had evidence of UC extending proximal to the rectum [ie, not limited to proctitis] or evidence of CD involving the ileum and/or colon, at a minimum.
6. Patients with extensive colitis or pancolitis of >8 years duration or left-sided colitis of >12 years duration must have had documented evidence that a surveillance colonoscopy was performed within 12 months prior to their first dose of study drug.
7. Patients who were sexually active agreed to use adequate contraception throughout the duration of the study and for 18 weeks after last dose, or agreed to completely abstain from heterosexual intercourse.
8. Patients with a family history of colorectal cancer [ie, first-degree relative], personal history of increased colorectal cancer risk or other known risk factor were up to date on colorectal cancer surveillance.
9. Vaccinations were up to date.
10. The patient had an inadequate response, loss of response or intolerance to at least one of the following agents, as defined below:

**Corticosteroids**

- 1. Signs and/or symptoms of persistently active disease despite a history of at least one 4-week induction regimen that included a dose equivalent to or more than 1 mg/kg daily prednisone orally for 2 weeks or intravenous [IV] for 1 week.

OR

- 1. Two failed attempts to taper corticosteroids to below a dose equivalent to 10 mg daily prednisone orally on two separate occasions.

OR

- 1. History of significant intolerance to corticosteroids [including, but not limited to, Cushing’s syndrome, osteopenia/osteoporosis, hyperglycaemia, insomnia and infection].

**Immunomodulators**

1. Signs and symptoms of persistently active disease despite a history of at least one 8-week regimen of oral azathioprine [≥1.5 mg/kg/day] or mercaptopurine mg/kg [≥1.0 mg/kg/day] or methotrexate [≥10 mg/m^2^ once a week].

OR

1. History of intolerance to at least one immunomodulator [including, but not limited to, nausea/vomiting, abdominal pain, pancreatitis, liver function test abnormalities, lymphopenia, thiopurine methyltransferase genetic mutation and infection].

**Tumour necrosis factor antagonists**

1. Signs and symptoms of persistently active disease despite a history of at least one induction regimen of infliximab 5 mg/kg IV at Weeks 0, 2 and 6, or adalimumab 2-week regimen of 160 mg on Day 1 and 80 mg on Day 15 if body weight was ≥40 kg, or 80 mg on Day 1 and 40 mg on Day 15 if body weight was <40 kg. For any other tumour necrosis factor antagonist, the patient must demonstrate signs and symptoms of persistently active disease despite a history of at least one induction regimen, as determined by the investigator.

OR

1. Recurrence of symptoms during maintenance dosing following prior clinical benefit, ie, fitting clinically with secondary loss of response [discontinuation despite clinical benefit does not qualify].

OR

1. History of intolerance to infliximab or adalimumab [including, but not limited to, infusion-related reaction, demyelination, congestive heart failure and infection].
2. The patient may have received the following drugs:
3. Oral 5-aminosalicylic acid compounds, provided the dose was stable for the 2 weeks prior to first dose of study drug.
4. Oral corticosteroid therapy [prednisone at a stable dose of ≤50 mg/day, or equivalent steroid], provided that the dose was stable for the 4 weeks prior to first dose of study drug if corticosteroids have been initiated, or for the 2 weeks prior to first dose of study drug if corticosteroids are being tapered.
5. Probiotics [eg, *Saccharomyces boulardii*], provided the dose was stable for the 2 weeks prior to first dose of study drug.
6. Antidiarrhoeals [eg, loperamide, diphenoxylate with atropine] for control of chronic diarrhoea.
7. Antibiotics used for the treatment of CD [eg, ciprofloxacin, metronidazole], provided the dose was stable for the 2 weeks prior to first dose of study drug.
8. Azathioprine or mercaptopurine, provided the dose was stable for the 8 weeks prior to first dose of study drug.
9. Methotrexate, provided the dose was stable for the 8 weeks prior to first dose of study drug.

***Exclusion criteria***

Any patient who met any of the following criteria did not qualify for entry into the study:

1. The patient had previous exposure to approved or investigational anti-integrins including, but not limited to, natalizumab, efalizumab, etrolizumab, or AMG 181, MAdCAM-1 antagonists or rituximab.
2. The patient had prior exposure to vedolizumab.
3. The patient had hypersensitivity or allergies to any of the vedolizumab excipients.
4. The patient had received:
5. Any investigational biologic [other than those listed in Exclusion criterion #1] within 60 days or 5 half-lives prior to screening [whichever is longer].
6. An approved biologic or biosimilar agent within 2 weeks prior to the first dose of the study drug or at any time during the screening period.
7. The patient had a positive progressive multifocal leukoencephalopathy subjective symptom checklist prior to the administration of the first dose of study drug.
8. The patient required surgical intervention for UC or CD, or was anticipated to require surgical intervention for UC or CD during this study.
9. Within 30 days prior to first dose of study drug, the patient received any of the following for the treatment of underlying disease:
10. Non-biologic therapies [eg, cyclosporine, thalidomide] other than those specifically listed in the protocol.
11. A non-biologic investigational therapy.
12. Use of topical [rectal] treatment with 5-aminosalicylic acid or corticosteroid within 2 weeks of the administration of the first dose of study drug.
13. The patient had, in the judgment of the investigator, clinically significant abnormal haematological parameters of haemoglobin, haematocrit or erythrocytes at screening.
14. The patient had any of the following laboratory abnormalities during the screening period:
15. Lymphocyte count <1.0 × 109/L or investigator concern regarding underlying lymphocytopenia.
16. Alanine aminotransferase or aspartate aminotransferase >3 × the upper limit of normal [ULN].
17. Alkaline phosphatase >3 × ULN.
18. Serum creatinine >2 × ULN.
19. The patient had any unstable or uncontrolled cardiovascular, heart failure moderate to severe [New York Class Association III or IV], pulmonary, hepatic, renal, gastrointestinal, genitourinary, haematological, coagulation, immunological, endocrine/metabolic, neurological or other medical disorder that, in the opinion of the investigator, would confound the study results or compromise patient safety.
20. Active or latent tuberculosis [TB], as evidenced by a diagnostic TB test performed within 30 days of screening or during the screening period that is positive, defined as:
21. Positive QuantiFERON test or two successive indeterminate QuantiFERON tests, OR
22. A TB skin test reaction ≥5 mm.
23. Clinically significant current or recent history [within 1 year prior to signing of informed consent/assent] of alcohol dependence or illicit drug use.
24. If the patient was required to take excluded medications.
25. If female, if the patient was pregnant or lactating or intending to become pregnant before, during or within 18 weeks after the last dose of study drug, or intending to donate ova during such time period. If male, if the patient was intending to donate sperm during the course of this study or for 18 weeks after the last dose of study drug.
26. The patient had a current diagnosis of indeterminate colitis. For patients less than 6 years of age, any findings that suggested monogenic very early onset inflammatory bowel disease were excluded.
27. The patient had evidence of abdominal abscess or toxic megacolon at the screening visit.
28. The patient had ileostomy, colostomy, ileo-anal pouch or known fixed symptomatic stenosis of the intestine.
29. The patient had extensive colonic resection, eg, subtotal or total colectomy.
30. The patient had a history or evidence of adenomatous colonic polyps that have not been removed.
31. The patient had a history or evidence of colonic mucosal dysplasia.
32. The patient had chronic hepatitis B virus [HBV] infection or chronic hepatitis C virus infection. However, HBV immune patients [ie, hepatitis B surface antigen–negative and hepatitis B antibody–positive] could be included.
33. The patient had any identified congenital or acquired immunodeficiency [eg, common variable immunodeficiency, human immunodeficiency virus infection, organ transplantation].
34. The patient had evidence of or treatment for *Clostridium difficile* infection within 60 days or other intestinal pathogen within 30 days prior to first dose of study drug.
35. The patient had other serious comorbidities that will limit his or her ability to complete the study.
36. The patient had any history of malignancy, except for the following: [a] adequately treated non-metastatic basal cell skin cancer; [b] squamous cell skin cancer that had been adequately treated and that has not recurred for at least 1 year prior to enrolment; and [c] history of cervical carcinoma in situ that had been adequately treated and that had not recurred for at least 3 years prior to first dose of study drug. Patients with remote history of malignancy [eg, >10 years since completion of curative therapy without recurrence] were considered on the basis of the nature of the malignancy and the therapy received, and inclusion was discussed with the sponsor on a case-by-case basis prior to enrolment.
37. The patient had a history of any major neurological disorders, including stroke, multiple sclerosis, brain tumour or neurodegenerative disease.
38. The patient had active psychiatric problems that, in the investigator’s opinion, may interfere with compliance with the study procedures. This included affective disorders that may confound the interpretation of patient reporting of gastrointestinal symptoms [eg, abdominal pain] in the opinion of the investigator.
39. The patient had a clinically significant infection [eg, pneumonia, pyelonephritis] within 30 days prior to first dose of study drug.
40. The patient had received any live vaccinations within 30 days prior to first dose of study drug.
41. The patient had history of lupus.
42. The patient had a surgical procedure requiring general anaesthesia within 30 days prior to screening or was planning to undergo major surgery during the study period.
43. The patient was unable to comply with all study assessments.
44. The patient was an immediate family member or study-site employee, or was in a dependent relationship with a study-site employee who was involved in the conduct of this study [eg, spouse, parent, child, sibling], or may consent under duress.

**Permitted Medications**

The following medications were permitted during the study:

- Immunomodulators [such as methotrexate, azathioprine, 6-mercaptopurine], stable for at least 8 weeks before first study drug dose.
- Oral 5-aminosalicylic acid, probiotics, enteral nutrition or antibiotics for the treatment of UC or CD that stable for at least 2 weeks before first study drug dose.
- Antidiarrheals for control of chronic diarrhoea. Any significant increase in the patient’s use in the 2 weeks before first study drug dose had to be considered by the investigator.

Concomitant medications, with the exception of corticosteroids [CS], were maintained at a constant dose throughout the first 14 weeks of the study. Initiation of CS, immunosuppressants or other therapies was not permitted during the study. CS was permitted at a maximum dose of ≤50 mg/day of prednisone [or equivalent steroid] and could be maintained at no more than this maximum permitted dose or could be tapered using a specific protocol [see below].

**Oral Corticosteroid Tapering**

***Screening and baseline/week 0 [first dose of study drug]***

CS doses [at maximum dose of 50 mg/day] taken during the screening could be tapered off up until the final 2 weeks before the first study drug dose when a stable CS dose had to be maintained. Patients who continued to receive CS at baseline, had to remain on the stable dose for at least a further 2 weeks after their first study drug dose, for a total of 4 weeks of stable CS dose.

***Week 2 [second dose of study drug]***

After the Week 2 study drug dose, CS dose tapering had to be initiated at the discretion of the investigator:

- For patients entering the study at a CS dose of ≥20 mg/day, doses were reduced by 5 mg/week until 20 mg/day for patients weighing ≥40 kg, and until 0.5 mg/kg/day for those weighing <40 kg.
- For patients entering the study at a CS dose of <20 mg/day, doses were reduced by 5 mg/week until 10 mg/day for patients weighing ≥40 kg and until 0.25 mg/kg/day for those weighing <40 kg.

Once these thresholds were reached, the CS dose was held stable until the third study drug dose was given at Week 6.

***Week 6 and 14 [third and fourth doses of study drug]***

Thereafter, between Weeks 6 and 14, CS tapering occurred at the discretion of the investigator by 5 mg/week until 10 mg/day, and thereafter by 2.5 mg/week until zero.

**Other exploratory efficacy endpoints for clinical response and remission**

- Proportion of patients with UC who achieve clinical response defined as a ≥20-point decrease from baseline in PUCAI score
- Proportion of patients with CD who achieve clinical response defined as a ≥15-point decrease from baseline in PCDAI score, and a maximum total PCDAI score of ≤30
- Proportion of patients with CD who achieved clinical response at Week 14 defined as a ≥50% reduction in SES-CD score from baseline or SES-CD score of 0–2 plus decrease in average daily abdominal pain score [CDAI component] of >0.25
- Proportion of patients with UC who achieve clinical remission at Week 14 defined as a complete Mayo score of ≤2 points with no individual subscore of >1
- Proportion of patients with CD who achieved clinical remission defined as a CDAI score of ≤150. Proportion of patients with UC who achieved a PUCAI score of <10
- Proportion of patients with CD who achieve a PCDAI score of ≤10 for CD.

**Supplementary Figure 1.** Study design. AE, adverse event; CD, Crohn’s disease; TNF, tumour necrosis factor; UC, ulcerative colitis; VDZ, vedolizumab, Wk, Week. ^a^Randomisation was stratified by previous exposure/failure to TNF antagonist therapy or naïvety to TNF antagonist therapy, by indication [UC or CD] and by weight group [≥30 kg or <30 kg]. ^b^Patients randomised to low-dose intravenous VDZ without any clinical response were escalated to high-dose VDZ. ^c^Patients with clinical response could exit and continue VDZ treatment in an extension study. ^d^Patients who withdrew before Wk 22 completed the Wk 22 assessments [and endoscopy if before the Wk 14 visit] at their early termination visit, and then attended the final safety visit 18 weeks after their last dose of VDZ.


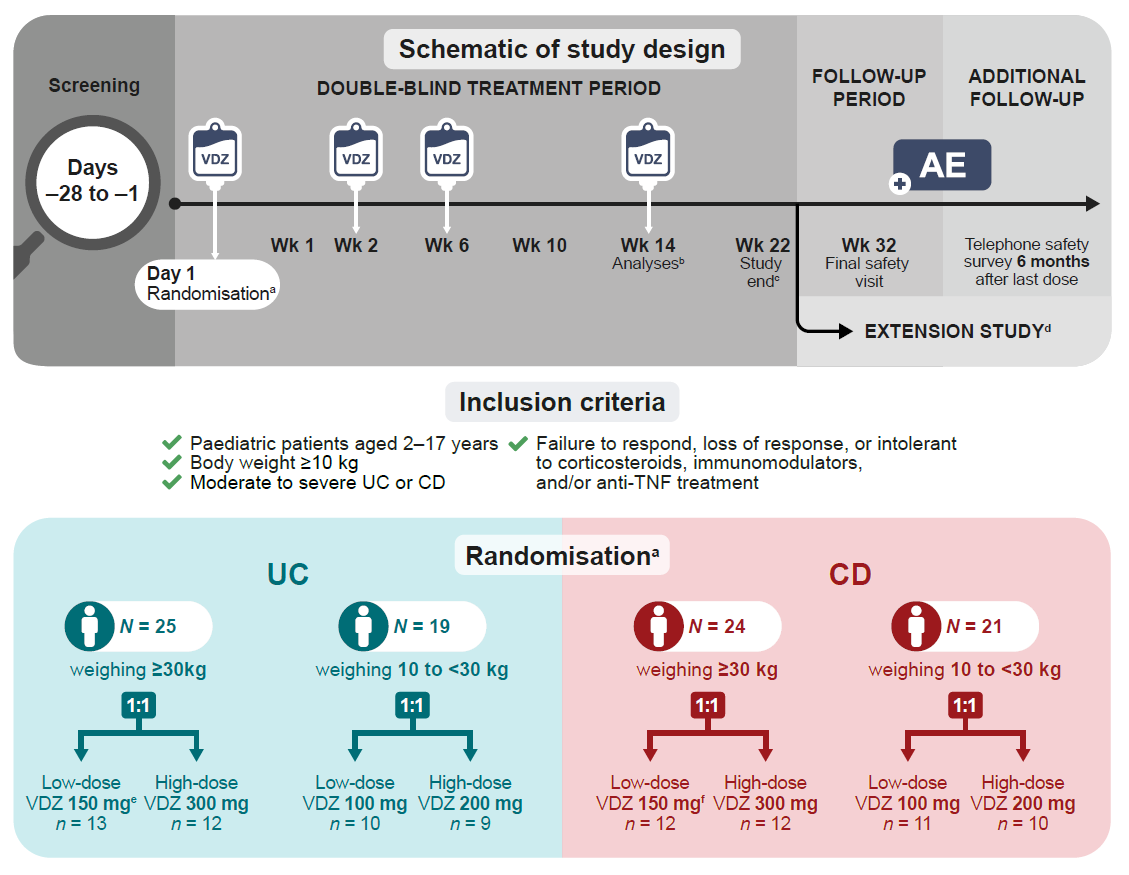


**Supplementary Table 1.** Enhanced clinical response rates among patients with CD [based on CDAI score^a^] at Week 14 [full analysis set].

|  | **CD** | | | |
| --- | --- | --- | --- | --- |
|  | **Body weight ≥30 kg [*n =* 23]** | | **Body weight <30 kg [*n =* 21]** | |
|  | **150 mg VDZ**  **[*n =* 11]** | **300 mg VDZ**  **[*n =* 12]** | **100 mg VDZ**  **[*n =* 11]** | **200 mg VDZ**  **[*n =* 10]** |
| Responder, *n* [%] | 4 [36.4] | 3 [25.0] | 7 [63.6] | 3 [30.0] |
| Jeffreys 95% CI^b^ | 34.8–86.3 | 47.1–92.4 | 34.8–86.3 | 9.3–60.6 |
| Exact 95% CI^c^ | 30.8–89.1 | 42.8–94.5 | 30.8–89.1 | 6.7–65.2 |
| Non-responder, *n* [%] | 7 [63.6] | 9 [75.0] | 4 [36.4] | 7 [70.0] |
| Jeffreys 95% CI^b^ | 13.7–65.2 | 7.6–52.9 | 13.7–65.2 | 39.4–90.7 |
| Exact 95% CI^c^ | 10.9–69.2 | 5.5–57.2 | 10.9–69.2 | 34.8–93.3 |

The full analysis set included all patients who received at least one dose of study drug according to treatment they were randomised to receive.

Any patient with missing data for determination of response status was counted as a non-responder.

CD, Crohn’s disease; CDAI, Crohn’s disease activity index; CI, confidence interval; VDZ, vedolizumab

^a^Clinical response, based on the CDAI score, was defined as a ≥100-point reduction from baseline in CDAI score. Patients with missing data at Week 14: body weight ≥30 kg [*n =* 1 in 150 mg VDZ, *n =* 2 in 300 mg VDZ]; Body weight <30 kg [*n =* 3 in 100 mg VDZ, *n =* 4 in 200 mg VDZ].

^b^Calculated using Jeffreys method. Jeffreys interval is a Bayesian credible interval obtained using the non-informative Jeffreys prior.

^c^The exact 95% CI was constructed on the basis of the Clopper-Pearson method.

**Supplementary Table 2.** Clinical response rates among patients with CD [based on SES-CD and CDAI scores^a^] at Week 14 [full analysis set].

|  | **CD** | | | |
| --- | --- | --- | --- | --- |
|  | **Body weight ≥30 kg  [*n =* 23]** | | **Body weight <30 kg  [*n =* 21]** | |
|  | **150 mg VDZ**  **[*n =* 11]** | **300 mg VDZ**  **[*n =* 12]** | **100 mg VDZ**  **[*n =* 11]** | **200 mg VDZ**  **[*n =* 10]** |
| Responder, *n* [%] | 1 [9.1] | 4 [33.3] | 4 [36.4] | 1 [10.0] |
| Jeffreys 95% CI^b^ | 1.0–35.3 | 12.5–61.2 | 13.7–65.2 | 1.1–38.1 |
| Exact 95% CI^c^ | 0.2–41.3 | 9.9–65.1 | 10.9–69.2 | 0.3–44.5 |
| Non-responder, *n* [%] | 10 [90.9] | 8 [66.7] | 7 [63.6] | 9 [90.0] |
| Jeffreys 95% CI^b^ | 64.7–99.0 | 38.8–87.5 | 34.8–86.3 | 61.9–98.9 |
| Exact 95% CI^c^ | 58.7–99.8 | 34.9–90.1 | 30.8–89.1 | 55.5–99.7 |

Any patient with missing data for determination of response status was counted as a non-responder.

CD, Crohn’s disease; CI, confidence interval; PCDAI, paediatric Crohn’s disease activity index; PUCAI, paediatric ulcerative colitis activity index; UC, ulcerative colitis; VDZ, vedolizumab

^a^Clinical response was defined as a ≥50% reduction in SES-CD score from baseline [or meets criteria for clinical remission based on SES-CD score of 0–2] with accompanying decrease in average daily abdominal pain score [CDAI component] by >0.25 Patients with missing data at Week 14: body weight ≥30 kg [*n =* 1 in 150 mg VDZ, *n =* 2 in 300 mg VDZ]; Body weight <30 kg [*n =* 4 in 100 mg VDZ, *n =* 4 in 200 mg VDZ].

^b^Calculated using Jeffreys method. Jeffreys interval is a Bayesian credible interval obtained using the non-informative Jeffreys prior.

^c^The exact 95% CI was constructed on the basis of the Clopper-Pearson method.

**Supplementary Table 3.** Clinical response rates among patients with UC or CD [based on complete PUCAI^a^ or PCDAI^b^ scores] at Week 14 [full analysis set].

|  | **UC** | | | | **CD** | | | |
| --- | --- | --- | --- | --- | --- | --- | --- | --- |
|  | **Body weight ≥30 kg  [*n =* 25]** | | **Body weight <30 kg  [*n =* 19]** | | **Body weight ≥30 kg  [*n =* 23]** | | **Body weight <30 kg  [*n =* 21]** | |
|  | **150 mg VDZ**  **[*n =* 13]** | **300 mg VDZ**  **[*n =* 12]** | **100 mg VDZ**  **[*n =* 10]** | **200 mg VDZ**  **[*n =* 9]** | **150 mg VDZ**  **[*n =* 11]** | **300 mg VDZ**  **[*n =* 12]** | **100 mg VDZ**  **[*n =* 11]** | **200 mg VDZ**  **[*n =* 10]** |
| Responder, *n* [%] | 9 [69.2] | 6 [50.0] | 8 [80.0] | 5 [55.6] | 5 [45.5] | 6 [50.0] | 6 [54.5] | 5 [50.0] |
| Jeffreys 95% CI^c^ | 42.3–88.6 | 24.3–75.7 | 49.7–95.6 | 25.4–82.7 | 20.0–73.0 | 24.3–75.7 | 27.0–80.0 | 22.4–77.6 |
| Exact 95% CI^d^ | 38.6–90.9 | 21.1–78.9 | 44.4–97.5 | 21.2–86.3 | 16.7–76.6 | 21.1–78.9 | 23.4–83.3 | 18.7–81.3 |
| Non-responder, *n* [%] | 4 [30.8] | 6 [50.0] | 2 [20.0] | 4 [44.4] | 6 [54.5] | 6 [50.0] | 5 [45.5] | 5 [50.0] |
| Jeffreys 95% CI^c^ | 11.4–57.7 | 24.3–75.7 | 4.4–50.3 | 17.3–74.6 | 27.0–80.0 | 24.3–75.7 | 20.0–73.0 | 22.4–77.6 |
| Exact 95% CI^d^ | 9.1–61.4 | 21.1–78.9 | 2.5–55.6 | 13.7–78.8 | 23.4–83.3 | 21.1–78.9 | 16.7–76.6 | 18.7–81.3 |

Any patient with missing data for determination of response status was counted as a non-responder.

CD, Crohn’s disease; CI, confidence interval; PCDAI, paediatric Crohn’s disease activity index; PUCAI, paediatric ulcerative colitis activity index; UC, ulcerative colitis; VDZ, vedolizumab

^a^Clinical response, based on the PUCAI score, was defined as a ≥20-point decrease from baseline in PUCAI score. Patients with missing data at Week 14: body weight ≥30 kg [*n =* 2 in 150 mg VDZ, *n =* 2 in 300 mg VDZ]; Body weight <30 kg [*n =* 1 in 100 mg VDZ, *n =* 1 in 200 mg VDZ].

^b^Clinical response, based on the PCDAI score, was defined as a ≥15-point decrease from baseline in PCDAI score, with a total PCDAI score of ≤30. Patients with missing data at Week 14: body weight ≥30 kg [*n =* 1 in 150 mg VDZ, *n =* 2 in 300 mg VDZ]; Body weight <30 kg [*n =* 3 in 100 mg VDZ, *n =* 3 in 200 mg VDZ].

^c^Calculated using Jeffreys method. Jeffreys interval is a Bayesian credible interval obtained using the non-informative Jeffreys prior.

^d^The exact 95% CI was constructed on the basis of the Clopper-Pearson method.

**Supplementary Table 4.** VDZ exposure at Week 14 according to clinical responsiveness [PUCAI or PCDAI; pharmacokinetic analysis set].

|  | **UC** | | | | **CD** | | | |
| --- | --- | --- | --- | --- | --- | --- | --- | --- |
|  | **Body weight ≥30 kg**  **[*n =* 25]** | | **Body weight <30 kg**  **[*n =* 19]** | | **Body weight ≥30 kg**  **[*n =* 23]** | | **Body weight <30 kg**  **[*n =* 21]** | |
|  | **150 mg VDZ**  **[*n =* 13]** | **300 mg VDZ**  **[*n =* 12]** | **100 mg VDZ**  **[*n =* 10]** | **200 mg VDZ**  **[*n =* 9]** | **150 mg VDZ**  **[*n =* 11]** | **300 mg VDZ**  **[*n =* 12]** | **100 mg VDZ**  **[*n =* 11]** | **200 mg VDZ**  **[*n =* 10]** |
| **C_trough_ Wk14 of responsive patients, µg/mL** | | | | | | | | |
| Patients, *n* | 9 | 6 | 7 | 4 | 5 | 6 | 6 | 5 |
| Median [IQR] | 11.4  [5.9–20.9] | 27.4  [8.6–40.6] | 9.5  [4.0–13.4] | 11.0  [8.9–22.0] | 3.0  [0.9–10.1] | 8.9  [0–13.0] | 4.0  [2.3–8.5] | 13.9  [1.7–24.2] |
| Range | 0–62.6 | 1.1–50.6 | 0–30.1 | 8.8–30.9 | 0–10.3 | 0–31.5 | 0.5–10.1 | 0–26.7 |
| Mean [SD] | 17.3 [18.6] | 25.9 [20.4] | 10.3 [9.8] | 15.4 [10.5] | 4.9 [5.0] | 10.4 [11.7] | 4.9 [3.7] | 13.3 [12.3] |
| **C_trough_ Wk14 of non-responsive patients, µg/mL** | | | | | | | | |
| Patients, *n* | 2 | 4 | 1 | 3 | 5 | 4 | 2 | 3 |
| Median [IQR] | 12.3  [6.8–17.8] | 13.7  [4.8–24.9] | 2.6  [2.6–2.6] | 0.5  [0–12.8] | 3.2  [2.8–4.1] | 3.2  [1.9–6.1] | 20.3  [19.8–20.7] | 0.3  [0–16.1] |
| Range | 6.8–17.8 | 0–31.9 | 2.6–2.6 | 0–12.8 | 0.4–4.2 | 1.7–7.8 | 19.8–20.7 | 0–16.1 |
| Mean [SD] | 12.3 [7.8] | 14.8 [13.5] | 2.6 [NA] | 4.4 [7.2] | 2.9 [1.5] | 4.0 [2.8] | 20.3 [0.6] | 5.5 [9.2] |

Exposure–response relationship was based on a ≥20-point decrease from baseline in PUCAI score for UC and a ≥15-point decrease from baseline in PCDAI score for CD, with total score of ≤30. The pharmacokinetic analysis set was defined as all patients who received at least one dose of VDZ and had at least one measurable concentration of VDZ.

CD, Crohn’s disease; IQR, interquartile range; NA, not available; PCDAI, paediatric Crohn’s disease activity index; PUCAI, paediatric ulcerative colitis activity index; SD, standard deviation; UC, ulcerative colitis; VDZ, vedolizumab.

**Supplementary Table 5.** Clinical remission rates among patients with UC or CD [based on complete Mayo^a^ or CDAI^b^ scores] at Week 14 [full analysis set].

|  | **UC** | | | | **CD** | | | |
| --- | --- | --- | --- | --- | --- | --- | --- | --- |
|  | **Body weight ≥30 kg  [*n =* 25]** | | **Body weight <30 kg  [*n =* 19]** | | **Body weight ≥30 kg  [*n =* 23]** | | **Body weight <30 kg  [*n =* 21]** | |
|  | **150 mg VDZ**  **[*n =* 13]** | **300 mg VDZ**  **[*n =* 12]** | **100 mg VDZ**  **[*n =* 10]** | **200 mg VDZ**  **[*n =* 9]** | **150 mg VDZ**  **[*n =* 11]** | **300 mg VDZ**  **[*n =* 12]** | **100 mg VDZ**  **[*n =* 11]** | **200 mg VDZ**  **[*n =* 10]** |
| Remitter, *n* [%] | 5 [38.5] | 4 [33.3] | 2 [20.0] | 2 [22.2] | 7 [63.6] | 6 [50.0] | 7 [63.6] | 5 [50.0] |
| Jeffreys 95% CI^c^ | 16.5–65.0 | 12.5–61.2 | 4.4–50.3 | 4.9–54.4 | 34.8–86.3 | 24.3–75.7 | 34.8–86.3 | 22.4–77.6 |
| Exact 95% CI^d^ | 13.9–68.4 | 9.9–65.1 | 2.5–55.6 | 2.8–60.0 | 30.8–89.1 | 21.1–78.9 | 30.8–89.1 | 18.7–81.3 |
| Non-remitter, *n* [%] | 8 [61.5] | 8 [66.7] | 8 [80.0] | 7 [77.8] | 4 [36.4] | 6 [50.0] | 4 [36.4] | 5 [50.0] |
| Jeffreys 95% CI^c^ | 35.0–83.5 | 38.8–87.5 | 49.7–95.6 | 45.6–95.1 | 13.7–65.2 | 24.3–75.7 | 13.7–65.2 | 22.4–77.6 |
| Exact 95% CI^d^ | 31.6–86.1 | 34.9–90.1 | 44.4–97.5 | 40.0–97.2 | 10.9–69.2 | 21.1–78.9 | 10.9–69.2 | 18.7–81.3 |

Any patients with missing data/component for determination of clinical remission status was considered a non-remitter. The full analysis set included all patients who received at least one dose of study drug according to treatment they were randomised to receive.

CD, Crohn’s disease; CDAI, Crohn’s disease activity index; CI, confidence interval; UC, ulcerative colitis; VDZ, vedolizumab

^a^Clinical remission in patients with UC was based on complete Mayo score of ≤2 points with no individual subscore of >1. Patients with missing data at Week 14: body weight ≥30 kg [*n =* 2 in 150 mg VDZ, *n =* 2 in 300 mg VDZ]; Body weight <30 kg [*n =* 3 in 100 mg VDZ, *n =* 1 in 200 mg VDZ].

^b^Clinical remission in patients with CD was based on CDAI score of ≤150. Patients with missing data at Week 14: body weight ≥30 kg [*n =* 1 in 150 mg VDZ, *n =* 2 in 300 mg VDZ]; Body weight <30 kg [*n =* 3 in 100 mg VDZ, *n =* 4 in 200 mg VDZ].

^c^Calculated using Jeffreys method. Jeffreys interval is a Bayesian credible interval obtained using the non-informative Jeffreys prior.

^d^The exact 95% CI was constructed on the basis of the Clopper-Pearson method.**Supplementary Table 6.** Clinical remission rates among patients with UC or CD [based on PUCAI^a^ or PCDAI^b^ score] at Week 14 [full analysis set].

|  | **UC** | | | | **CD** | | | |
| --- | --- | --- | --- | --- | --- | --- | --- | --- |
|  | **Body weight ≥30 kg  [*n =* 25]** | | **Body weight <30 kg  [*n =* 19]** | | **Body weight ≥30 kg  [*n =* 23]** | | **Body weight <30 kg  [*n =* 21]** | |
|  | **150 mg VDZ**  **[*n =* 13]** | **300 mg VDZ**  **[*n =* 12]** | **100 mg VDZ**  **[*n =* 10]** | **200 mg VDZ**  **[*n =* 9]** | **150 mg VDZ**  **[*n =* 11]** | **300 mg VDZ**  **[*n =* 12]** | **100 mg VDZ**  **[*n =* 11]** | **200 mg VDZ**  **[*n =* 10]** |
| Remitter, *n* [%] | 8 [61.5] | 5 [41.7] | 3 [30.0] | 4 [44.4] | 5 [45.5] | 2 [16.7] | 6 [54.5] | 5 [50.0] |
| Jeffreys 95% CI^c^ | 35.0–83.5 | 18.0–68.8 | 9.3–60.6 | 17.3–74.6 | 20.0–73.0 | 3.6–43.6 | 27.0–80.0 | 22.4–77.6 |
| Exact 95% CI^d^ | 31.6–86.1 | 15.2–72.3 | 6.7–65.2 | 13.7–78.8 | 16.7–76.6 | 2.1–48.4 | 23.4–83.3 | 18.7–81.3 |
| Non-remitter, *n* [%] | 5 [38.5] | 7 [58.3] | 7 [70.0] | 5 [55.6] | 6 [54.5] | 10 [83.3] | 5 [45.5] | 5 [50.0] |
| Jeffreys 95% CI^c^ | 16.5–65.0 | 31.2–82.0 | 39.4–90.7 | 25.4–82.7 | 27.0–80.0 | 56.4–96.4 | 20.0–73.0 | 22.4–77.6 |
| Exact 95% CI^d^ | 13.9–68.4 | 27.7–84.8 | 34.8–93.3 | 21.2–86.3 | 23.4–83.3 | 51.6–97.9 | 16.7–76.6 | 18.7–81.3 |

Any patients with missing data/component for determination of clinical remission status was considered a non-remitter. The full analysis set included all patients who received at least one dose of study drug according to treatment they were randomised to receive.

CD, Crohn’s disease; CI, confidence interval; PCDAI, paediatric Crohn’s disease activity index; PUCAI, paediatric ulcerative colitis activity index; UC, ulcerative colitis; VDZ, vedolizumab

^a^Clinical remission in patients with UC was based on PUCAI score of <10. Patients with missing data at Week 14: body weight ≥30 kg [*n =* 2 in 150 mg VDZ, *n =* 2 in 300 mg VDZ]; Body weight <30 kg [*n =* 1 in 100 mg VDZ, *n =* 1 in 200 mg VDZ].

^b^Clinical remission in patients with CD was based on PCDAI score of ≤10. Patients with missing data at Week 14: body weight ≥30 kg [*n =* 1 in 150 mg VDZ, *n =* 2 in 300 mg VDZ]; Body weight <30 kg [*n =* 3 in 100 mg VDZ, *n =* 3 in 200 mg VDZ].

^c^Calculated using Jeffreys method. Jeffreys interval is a Bayesian credible interval obtained using the non-informative Jeffreys prior.

^d^The exact 95% CI was constructed on the basis of the Clopper-Pearson method.

**Supplementary Table 7.** Baseline faecal calprotectin [μg/g] and change from baseline to Week 14 [full analysis set].

|  | **UC** | | | | **CD** | | | |
| --- | --- | --- | --- | --- | --- | --- | --- | --- |
|  | **Body weight ≥30 kg  [*n =* 25]** | | **Body weight <30 kg  [*n =* 19]** | | **Body weight ≥30 kg  [*n =* 23]** | | **Body weight <30 kg  [*n =* 21]** | |
|  | **150 mg VDZ**  **[*n =* 13]** | **300 mg VDZ**  **[*n =* 12]** | **100 mg VDZ**  **[*n =* 10]** | **200 mg VDZ**  **[*n =* 9]** | **150 mg VDZ**  **[*n =* 11]** | **300 mg VDZ**  **[*n =* 12]** | **100 mg VDZ**  **[*n =* 11]** | **200 mg VDZ**  **[*n =* 10]** |
| Baseline | | | | | | | | |
| Patients, *n* | 13 | 11 | 10 | 9 | 10 | 12 | 11 | 10 |
| Mean [SD], μg/g | 3364 [3316] | 1335 [921] | 1714 [1709] | 3097 [3377] | 2760 [3565] | 1443 [837] | 1666 [1217] | 4014 [5943] |
| Median [range], μg/g | 2517  [410– 10,654] | 1466  [147–2996] | 1194  [48–5546] | 1824  [10–10,685] | 1258  [408–10,737] | 1421  [22–2922] | 1495  [483-4578] | 1155  [162–18,978] |
| Change from baseline | | | | | | | | |
| Patients, *n* | 11 | 10 | 8 | 8 | 9 | 11 | 7 | 7 |
| Mean [SD], μg/g | -1985 [2923] | -4 [1928] | 1560 [5480] | -1549 [4470] | -374 [3203] | 992 [4571] | -1111 [1739] | -1571 [3765] |
| Median [range], μg/g | -1527  [-8897–1864] | -351  [-2318–3481] | 1239  [-5506–13,659] | -632  [-10,393–3193] | -158  [-7707–4127] | -398  [-1535–14,659] | -692  [-4239–1458] | -613  [-9618–1424] |

**Supplementary Table 8.** All AEs that occurred in ≥10% of total patients with UC or CD [per weight group and indication] who received intravenous vedolizumab [safety analysis set].

| **System Organ Class**  **High Level Term**  **Preferred Term^a^** | **Body weight ≥30 kg [*n =* 48]** | | **Body weight <30 kg [*n =* 40]** | |
| --- | --- | --- | --- | --- |
|  | **UC**  **[*n =* 25]** | **CD**  **[*n =* 23]** | **UC**  **[*n =* 19]** | **CD**  **[*n =* 21]** |
| **Patients with any AEs, *n* [%]** | 20 [80.0] | 21 [91.3] | 16 [84.2] | 19 [90.5] |
| **Gastrointestinal disorders, *n* [%]** | 9 [36.0] | 11 [47.8] | 5 [26.3] | 11 [52.4] |
| Gastrointestinal and abdominal pains (excluding oral and throat) | <10% | <10% | 4 [21.1] | 7 [33.3] |
| Abdominal pain | <10% | <10% | 4 [21.1] | 6 [28.6] |
| Colitis (excludes infective) | 5 [20.0] | 5 [21.7] | 3 [15.8] | 6 [28.6] |
| Colitis ulcerative | 5 [20.0] | – | 3 [15.8] | – |
| Crohn’s disease | – | 5 [21.7] | – | 5 [23.8] |
| Nausea and vomiting symptoms | <10% | <10% | <10% | 4 [19.0] |
| Vomiting | <10% | – | <10% | 3 [14.3] |
| Gastrointestinal atonic and hypomotility disorders NEC | – | 3 [13.0] | – | – |
| **Infections and infestations, *n* [%]** | 12 [48.0] | 11 [47.8] | 7 [36.8] | 6 [28.6] |
| Upper respiratory tract infections | 4 [16.0] | 5 [21.7] | 3 [15.8] | <10% |
| Upper respiratory tract infection | <10% | <10% | 2 [10.5] | – |
| Viral infection NEC | <10% | 3 [13.0] | 3 [15.8] | <10% |
| Respiratory tract viral infection | – | – | 2 [10.5] | – |
| Viral infection | <10% | 3 [13.0] | <10% | <10% |
| Abdominal and gastrointestinal infections | – | 3 [13.0] | – | – |
| Lower respiratory tract and lung infections | 3 [12.0] | – | <10% | <10% |
| **Skin and subcutaneous tissue disorders, *n* [%]** | <10% | 7 [30.4] | – | 4 [19.0] |
| **Respiratory, thoracic and mediastinal disorders, *n* [%]** | <10% | 6 [26.1] | 2 [10.5] | 5 [23.8] |
| Upper respiratory tract signs and symptoms | – | 4 [17.4] | 2 [10.5] | 4 [19.0] |
| Oropharyngeal pain | – | <10% | <10% | 3 [14.3] |
| **Metabolism and nutrition disorders, *n* [%]** | – | 6 [26.1] | – | <10% |
| Iron deficiencies (iron deficiency) | – | 3 [13.0] | – | – |
| **Investigations, *n* [%]** | 3 [12.0] | <10% | 3 [15.8] | 5 [23.8] |
| Red blood cell analyses (decreased haemoglobin) | 3 [12.0] | – | <10% | – |
| Protein analyses NEC | <10% | – | 2 [10.5] | <10% |
| **Nervous system disorders, *n* [%]** | 3 [12.0] | 5 [21.7] | 2 [10.5] | <10% |
| Headache NEC (headache) | <10% | 5 [21.7] | 2 [10.5] | <10% |
| **Blood and lymphatic system disorders, *n* [%]** | <10% | 3 [13.0] | 4 [21.1] | 4 [19.0] |
| Anaemias NEC (anaemia) | <10% | <10% | 4 [21.1] | <10% |
| **Injury, poisoning and procedural complications, *n* [%]** | – | <10% | 4 [21.1] | <10% |
| **General disorders and administration site conditions, *n* [%]** | <10% | 4 [17.4] | 2 [10.5] | 4 [19.0] |
| Febrile disorders (pyrexia) | – | 3 [13.0] | <10% | 3 [14.3] |
| **Musculoskeletal and connective tissue disorders, *n* [%]** | 4 [16.0] | 4 [17.4] | 2 [10.5] | 3 [14.3] |
| Joint-related signs and symptoms (arthralgia) | – | 3 [13.0] | <10% | <10% |

The following AEs are not listed in the table because they occurred in <10% across both disease indications: disorders of the renal and urinary system; immune system; reproductive system and breast; ear and labyrinth; endocrine system; eye; neoplasms benign, malignant and unspecified [including cysts and polyps].

AE, adverse event; CD, Crohn’s disease; NEC, not elsewhere classified; UC, ulcerative colitis; VDZ, vedolizumab

^a^Preferred Terms are listed in parentheses in which the number of patients was the same as in the High Level Term.
