# Supplementary figures and images for: Pharmacokinetics, Safety and Efficacy of Intravenous Vedolizumab in Paediatric Patients with Ulcerative Colitis or Crohn’s Disease: Results from the Phase 2 HUBBLE Study
Source: J Crohns Colitis. 2022 Mar 17;16(8):1243–54. doi: 10.1093/ecco-jcc/jjac036 (PMC9426668; doi:10.1093/ecco-jcc/jjac036)

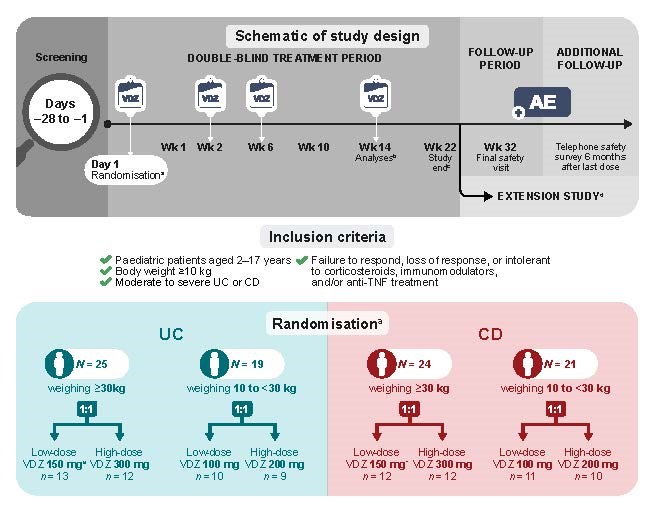

Supplement: jjac036_suppl_Supplementary_Figure_S1 [file jjac036_suppl_supplementary_figure_s1.jpeg]
